# *Toxoplasma gondii* seroprevalence in wild boars (*Sus scrofa*) in Sweden and evaluation of ELISA test performance

**DOI:** 10.1017/S0950268814002891

**Published:** 2014-11-06

**Authors:** C. WALLANDER, J. FRÖSSLING, I. VÅGSHOLM, A. UGGLA, A. LUNDÉN

**Affiliations:** 1 Department of Biomedical Sciences and Veterinary Public Health, Swedish University of Agricultural Sciences, Uppsala, Sweden; 2 Department of Disease Control and Epidemiology, National Veterinary Institute, Uppsala, Sweden; 3 Department of Animal Environment and Health, Swedish University of Agricultural Sciences, Skara, Sweden; 4 Department of Virology, Immunobiology and Parasitology, National Veterinary Institute, Uppsala, Sweden

**Keywords:** Monitoring, serological screening, test evaluation, *Toxoplasma gondii*, wild boar

## Abstract

*Toxoplasma gondii* is a zoonotic protozoan parasite, infecting a wide range of warm-blooded animals. The Swedish wild boar population is expanding and increased hunting provides its meat to a growing group of consumers. We performed a spatio-temporal investigation of *T. gondii* seroprevalence in Swedish wild boars. An ELISA was set up and evaluated against a commercial direct agglutination test, using Bayesian latent class analysis. The ELISA sensitivity and specificity were estimated to 79% and 85%, respectively. Of 1327 serum samples, 50% were positive. Thirty-four per cent of young wild boars and 55% of adults were positive (*P* < 0·001). The total seroprevalence ranged from 72% in 2005 to 38% in 2011 (*P* < 0·001), suggesting a declining trend. The highest seroprevalence, 65%, was recorded in South Sweden. In other regions it varied from 29% in Stockholm to 46% in East Middle Sweden.

## INTRODUCTION

*Toxoplasma gondii* is a zoonotic, potentially lethal, protozoan parasite with the ability to infect most warm-blooded animals, including humans. Infection occurs by ingesting oocysts, excreted into the environment with the faeces from newly infected cats (the main host of the parasite) or by consuming meat from previously infected intermediate host animals harbouring tissue cysts [1]. Recently, several studies have estimated *T. gondii* to be one of the most important pathogens involved in foodborne infections, causing a considerable disease burden and economic losses [2, 3]. The European Food Safety Authority (EFSA) has recommended the implementation of pre-harvest monitoring of *T. gondii* in sheep, goats, pigs, and game [4]. In Sweden, studies have been performed on *T. gondii* prevalence in different wildlife species such as brown hares (*Lepus europaeus* P.) [5], red foxes (*Vulpes vulpes*) [6], moose (*Alces alces*), roe deer (*Capreolus capreolus*) [7] and lynx (*Lynx lynx*) [8]. Wild boars (*Sus scrofa*) are located mainly in the southeastern part of the country but are, according to the Swedish Association for Hunting and Wildlife Management, rapidly and successively spreading north [9]. The destructive behaviour of wild boars in agricultural areas has demanded increased hunting and, as a result, wild boar meat is now available for a larger group of consumers. The prevalence of *T. gondii* in wild boars in Sweden has not been previously investigated. However, in nearby parts of Europe, studies have indicated seroprevalence rates at high levels, ranging from 25% to 35% [10–12]. Two of the most widely used methods for seroprevalence studies of *T. gondii* are direct agglutination tests (DAT) and enzyme-linked immunosorbent assays (ELISA) [13], the latter being practical and comparatively inexpensive if set up as an in-house system. It is essential that new tests are validated against a gold standard test and/or using sera from a population of animals with known disease status. However, test evaluation is also possible using, for example, a Bayesian statistical approach [14], which has been used in veterinary diagnostic test evaluations in recent years (e.g. see [15] and [16]). To assess the importance of wild boar meat as a source of *T. gondii* infection, the aims of this study were to: (*a*) evaluate the test performance of an in-house ELISA against a commercially available DAT, (*b*) estimate the prevalence of *T. gondii* antibodies in wild boars in Sweden based on submitted samples, and (*c*) investigate possible variations in seroprevalence due to age, region and year.

## METHODS

### Background population and study sample

**Fig. 1.**
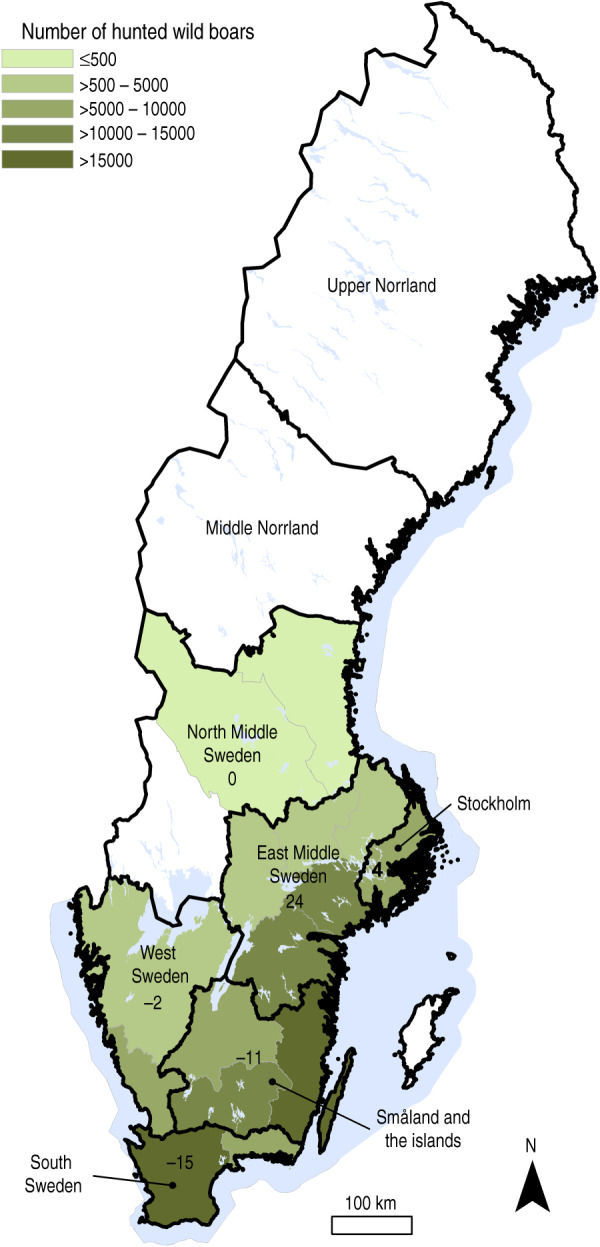
Geographical distribution of hunted wild boars in Sweden during the hunting seasons of 2009/2010 and 2010/2011. Based on data provided by the Swedish Association for Hunting and Wildlife Management, Wildlife Monitoring [9]. Colour scale represents number of animals hunted in each county (white areas represent no wild boars hunted). Regions (Nomenclature of Territorial Units for Statistics level 2) are drawn with black borders and black numbers on the map indicate percentage difference between proportion of hunted animals and proportion of sampled animals in each region. For example, South Sweden is underrepresented in our sample compared to the proportion of wild boars hunted in this region.

The wild boar population in Sweden increased to more than 100 000 wild boars by 2010/2011 (July–June) compared to ~40 000 animals in 2005/2006 [9]. The geographical distribution of wild boars has expanded during the study period from the southern area to include the middle-eastern parts of the country. The population distribution for 2009/2010 and 2010/2011 is shown in Figure 1. The material used in this study is a subset of samples from a national surveillance programme for absent diseases in Swedish wild boars [17]. This programme has been on-going since 2000 and is based on hunters submitting wild boar serum samples to the National Veterinary Institute (SVA) in Uppsala, Sweden. Information on geographical origin is available for most samples, and for samples collected in 2010 and onwards, information on age group based on phenotype (colour and size) is also registered. In this study the age groups were ⩽12 months and >12 months.

The samples analysed in this study were retrieved from samples collected in 2005 and from 2008 to 2011. The strategy was to randomly select 250 samples from each year. This sample size was based on a population size of 100 000–150 000 wild boars, a maximum expected seroprevalence of 25% [10–12] and an aim to detect a 10% change in seroprevalence with 95% confidence level and 80% power (Win Episcope 2·0 [18]). Between 10% and 30% of the samples were excluded due to poor quality; moreover, only a limited number of samples were available from 2005 and 2009. Finally, 1328 samples were included in the study and these represented six regions in Sweden (Nomenclature of Territorial Units for Statistics (NUTS) system, level 2 [19]) (Fig. 1). Information on animal age was available for 480 samples. The distribution of samples per year, age group and region, is shown in Table 1.

**Table 1.** Number and distribution of serum samples per year, age group (in months) and region

Variable	Category	2005	2008	2009	2010	2011	Total
Age	>12	n.a.	n.a.	n.a.	106	99	205
	⩽12	n.a.	n.a.	n.a.	132	143	275
	Unknown	n.a.	n.a.	n.a.	12	28	40
	Total	n.a.	n.a.	n.a.	250	270	520
Region	South Sweden	46	20	47	43 (23/18)*	12 (8/3)*	168
	Småland and the Islands	19	121	63	45 (25/20)*	51 (21/27)*	299
	West Sweden	33	105	43	27 (16/10)*	18 (17/1)*	226
	East Middle Sweden	67	118	87	107 (46/54)*	144 (70/60)*	523
	Stockholm	3	5	8	27 (21/4)*	28 (22/5)*	71
	North Middle Sweden	12	0	0	1 (1/0)*	0	13
	Unknown	10	0	0	0	17 (5/3)*	27
	Total	190	369	248	250	270	1327

n.a., Not applicable.

* Total number (⩽12 months/>12 months).

### Serology

#### ELISA

All serum samples were analysed using ELISA. This was based on a previously published assay for sheep sera utilizing a saponin-octylglucoside solubilized tachyzoite antigen from the RH strain of *T. gondii*, produced at the Moredun Research Institute, UK. The assay was performed as described previously [20] but with a peroxidase-labelled polyclonal rabbit anti-pig immunoglobulin G antibody (Sigma-Aldrich, USA) diluted 1: 10 000, Polysorp microtitre plates (Nunc Immuno Plate, Thermo Fisher Scientific, USA) and buffers and substrate from SVA. Washing was performed with a manual plate washer (Nunc-Immuno Wash 12, Thermo Fisher Scientific) and the optical density (OD) at 450 nm was read in a plate reader (Multiscan FC v. 2·5, Thermo Fisher Scientific). Positive and negative pig sera were used in checkerboard titrations to retrieve optimal dilutions for antigen, serum samples and conjugate, for which the ratio between OD values for positive and negative sera was as high as possible. The positive sera was provided by the National Veterinary Institute, Technical University of Denmark (Kgs. Lyngby, Denmark), and the negative sera were from fattening pigs in a closed herd (Serogrisen, Ransta, Sweden) which tested negative by DAT (described below) and immunoblotting, performed as described previously [21]. Control for plate-to-plate variation was performed as described previously [20], using seven controls, ranging in OD value from 0·10 to 1·52. A total of four ELISA plates were retested based on the calculated *R*^2^ (<0·97) [20]. Further, 25 individual samples were retested because of a coefficient of variation (CV; i.e. standard deviation of samples/mean value of samples) >20% between the duplicates. One sample did not manage to obtain a CV <20% in spite of multiple analyses and was excluded from the dataset.

#### DAT

To allow estimation of diagnostic sensitivity (Se) and specificity (Sp) of the ELISA on wild boar serum samples, a subset of the samples (*n* = 242) from 2011 was also analysed using a commercially available DAT, in which IgM-mediated agglutination is prevented by addition of 2-mercaptoethanol (Toxo-Screen DA, bioMérieux SA, France). The DAT was performed according to the manufacturer's instructions except that a serum dilution of 1:20 was used as positive cut-off. This cut-off has previously been evaluated for pig sera [22]. Samples were also tested in dilution 1:4000 to account for the prozone effect [23]. Sera positive in either dilution were classified as *T. gondii* positive. Out of 89 DAT-positive samples, 24 were positive only in dilution 1:4000. Seventy per cent of these samples had ELISA OD values >0·8, which agrees well with the prozone effect being a result of high levels of specific antibodies.

### Estimation of diagnostic Se and Sp

The test performance of the ELISA was evaluated using Bayesian latent class analysis based on two dependent tests and two populations as described by Branscum *et al*. [24]. For evaluation purposes, a subset of samples from 2011 (*n* = 242) were grouped into subpopulation 1 [wild boars aged ⩽12 months (*n* = 143)] and subpopulation 2 [wild boars aged >12 months (*n* = 99)]. Based on numerous reports of an age-dependent *T. gondii* prevalence in a wide range of host species [11, 12], the prevalence in subpopulation 1 (π1), was expected to be lower compared to the prevalence of subpopulation 2 (π2). The latent class model was run using OpenBUGS 3·2·2 [25]. After a burn-in period of 5000 iterations, estimates were based on the next 50 000 iterations. Multiple chains (set to different starting values) for every estimate were checked for convergence and stability by assessing history plots [26]. Prior information on the parameters to be estimated was included in the model as beta distributions. The priors for the diagnostic Se and Sp of DAT (Se_DAT_/Sp_DAT_) were set to a mode and 5th percentile of 0·83 and 0·65, and 0·90 and 0·80, respectively. This corresponded to beta(17·86,4·45) and beta(42·57,5·62) and was based on previously published evaluations of the test [22, 24]. To get a rough prior estimate of the seroprevalence in the young and adult population (π1/π2) a commercial ELISA (ID Screen Toxoplasmosis Indirect Multi-species; IDvet Innovative Diagnostics, France) was used. The apparent seroprevalence was 27% (95th percentile = 35%) in the young population and 40% (95th percentile = 51%) in the adult population. Using these estimates, and the 95th percentiles in the confidence intervals as the 95th percentiles for the beta distributions, provided beta(27·57,72·85) and beta(23·60,34·91) as priors for π1/π2, respectively. For Se analysis purposes, the model was also run using uniform priors [beta(1,1)] for both DAT test performance and prevalences. A series of cut-off-values for the ELISA were tested in the analysis to identify a suitable cut-off for our purpose. The selected ELISA cut-off value was based on the results from the latent class analysis and with regard to the calculated maximal Youden's index (Youden's index = Se + Sp – 1) [27]. Using this cut-off, all 1327 samples were classified as positive or negative.

Data management and statistical analysis was performed using R 2·15·2 [28]. Differences between groups were tested using the Pearson's *χ*^2^ test.

## RESULTS

### Seroprevalence in wild boars

**Fig. 2.**
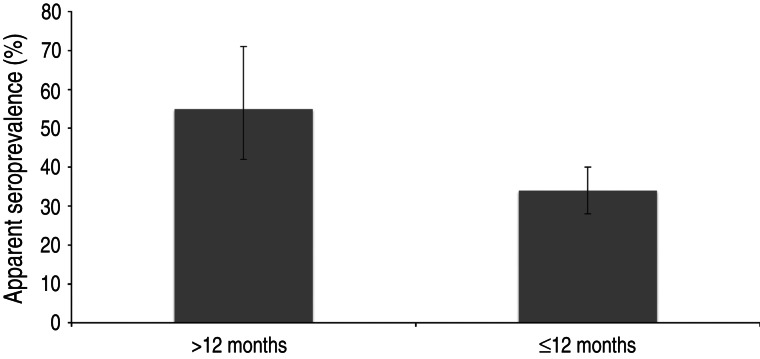
Apparent seroprevalence (with 95% confidence intervals) in animals aged ⩽12 months and >12 months in 2010–2012.

**Fig. 3.**
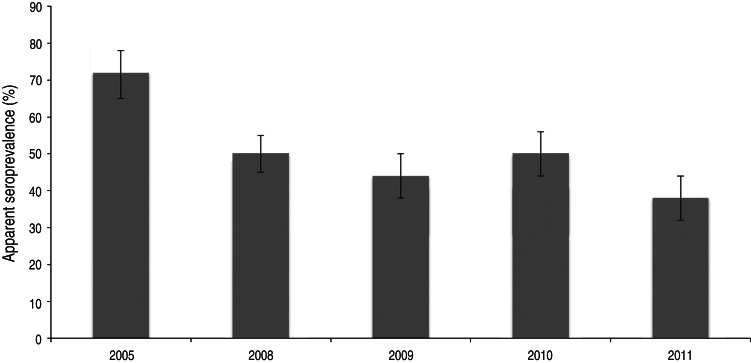
Apparent seroprevalence (with 95% confidence intervals) in different years.

**Fig. 4.**
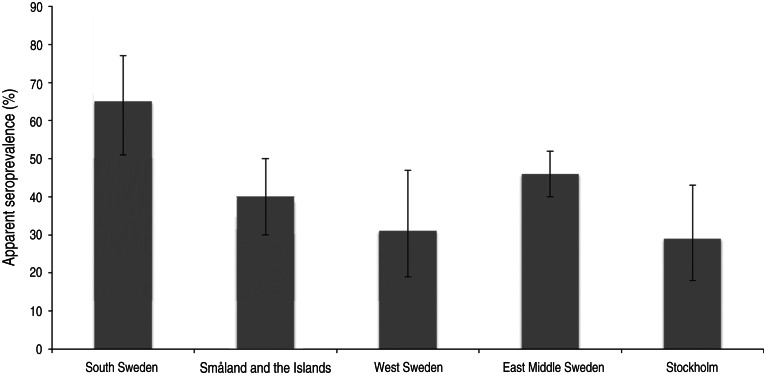
Apparent seroprevalence (with 95% confidence intervals) in different regions (Nomenclature of Territorial Units for Statistics level 2).

After classifying samples as positive or negative using the selected cut-off (see below), 657/1327 samples [50%; 95% confidence interval (CI) 47–52] scored positive. In total, 94/275 (34%, 95% CI 29–40) young wild boars and 113/205 (55%, 95% CI 48–62) adult wild boars were positive (Fig. 2). The difference in seroprevalence between the two age groups was significant (*P* < 0·001). The apparent seroprevalence per year varied between 38% (95% CI 32–44) in 2011 and 72% (95% CI 65–78) in 2005 (Fig. 3). The difference was significant between 2005 and 2011 (*P* < 0·001) and also between 2008 (50%, 95% CI 45–55) and 2011 (*P* < 0·05). Based on pair-wise comparisons, the apparent seroprevalence within different regions (considering only years 2010 and 2011 together, due to the rapidly changing population) was similar except for South Sweden, which had a significantly higher seroprevalence than all other regions (65%, 95% CI 51–77) (*P* < 0·05) (Fig. 4). The seroprevalence in other regions varied from 29% (95% CI 18–43) in Stockholm to 46% (95% CI 40–52) in East Middle Sweden.

### Test performance

**Fig. 5.**
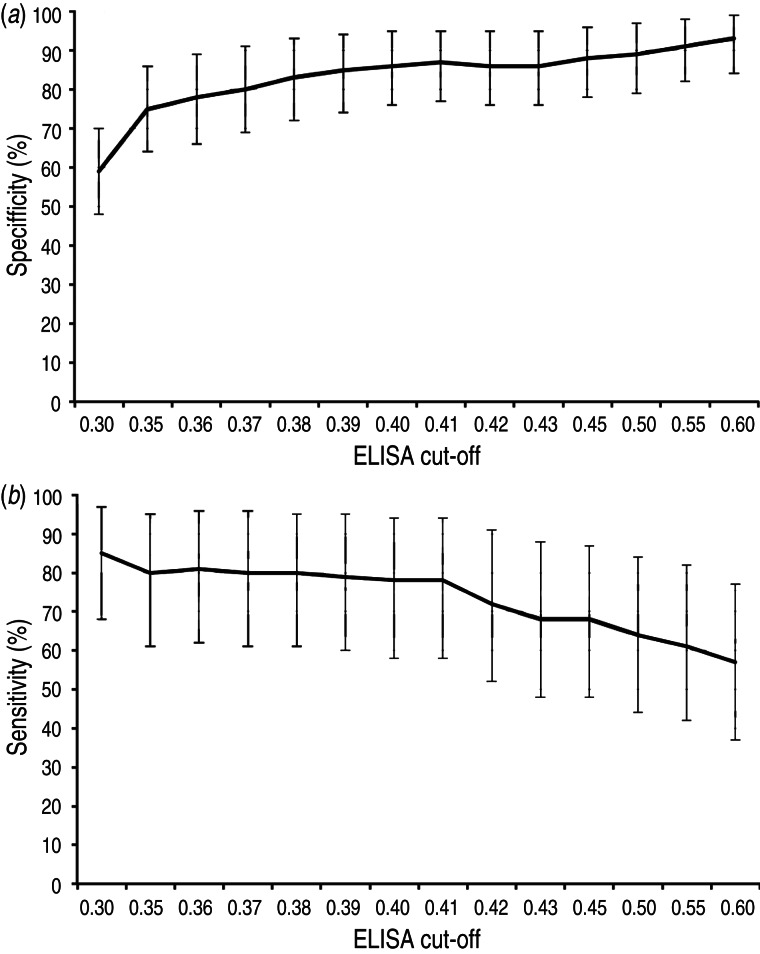
Estimated (*a*) specificity and (*b*) sensitivity (including 95% credibility interval) of the in-house ELISA at different cut-offs, based on the latent class analysis.

The estimated Se and Sp of the ELISA at different cut-offs, based on the latent class analysis, are presented in Figure 5. Of all cut-offs tested (0·30–0·60), cut-off values 0·35–0·41 showed the highest combined values for Se and Sp (Se = 80–78%, Sp = 75–87%). Lower and higher cut-offs displayed either too low Sp or too low Se for our purposes. Having a preference for a higher Se than Sp, the cut-off 0·39 was selected with Se = 79% [95% credibility interval (CrI) 60–95] and Sp = 85% (95% CrI 74–94). The cross-classified test results from the ELISA (cut-off 0·39) and the DAT on the 242 samples included in the latent class analysis are shown in Table 2. Including prior information in the model did not influence the posterior distribution of most parameters, except Se_DAT_ and π2. The results from the Se analysis are presented in Table 3.

**Table 2.** Cross-classified test results from the in-house ELISA (cut-off 0·39) and the DAT on the 242 samples included in the latent class analysis

Population 1 (⩽12 months)				Population 2 (>12 months)		
	ELISA+	ELISA−	Total	ELISA+	ELISA−	Total
DAT+	29	15	44	37	8	45
DAT-	13	86	99	10	44	54
Total	42	101	143	47	52	99

DAT, Direct agglutination test.

**Table 3.** Results of latent class sensitivity analysis, showing mean posterior estimate (95% credible interval) for all estimated parameters, when analysed using priors for DAT (Se_DAT_/Sp_DAT_), prevalences (π1/π2) and both

Estimated characteristic	Prior used		
	Se_DAT_/Sp_DAT_	π1/π2	Both
Se_ELISA_	76 (55–94)	76 (46–95)	79 (60–95)
Sp_ELISA_	85 (73–96)	82 (67–94)	85 (74–94)
Se_DAT_	79 (61–93)	77 (43–98)	82 (69–93)
Sp_DAT_	87 (78–95)	83 (66–96)	87 (80–94)
π1	27 (12–43)	26 (19–34)	27 (20–34)
π2	50 (33–72)	43 (33–54)	44 (34–59)

DAT, Direct agglutination test.

## DISCUSSION

This is the first study of *T. gondii* seroprevalence in wild boars in Sweden. Approximately half of the animals tested in this study were positive. Wild boar meat is becoming increasingly accessible in retail stores, and the modern consumer is not likely to be familiar with all possible health hazards associated with eating this meat undercooked. By analogy with the assumption that a large proportion of domestic pigs with antibodies to *T. gondii* also harbour viable tissue cysts [29], it is likely that people consuming wild boar meat in Sweden are at risk of contracting a *T. gondii* infection if the meat is ingested inadequately cooked.

Our finding of an age-dependent seroprevalence is in agreement with several other studies of *T. gondii* in wild boars [11, 12, 30]. However, not all studies have been able to confirm this [31]. Moreover, in a recent paper by Opsteegh and co-workers [10], it was suggested that seropositive wild boars might seroconvert back to negative. This was based on the observation that seroprevalence increased with age, but reached a plateau at around age 10 months. Moreover, their data fitted best into a disease spread model with conversion back to susceptibility for previously infected animals. This hypothesis implies that infected wild boars might be able to reduce or eliminate the infection. Thus, further research addressing the relationship between seropositivity and presence of tissue cysts in wild boars, and possibly also in domestic pigs, is needed for a quantitative food safety risk assessment.

The apparent seroprevalence varied between years and it was significantly higher in the first year studied than in the later years. This could reflect decreased contamination with oocysts in the environment, which is believed to be the most important source of infection for wild boars. However, since the age of animals sampled in the earlier years (2005–2009) were not known, the result could be due to a higher proportion of older animals in those years. In addition, the seroprevalence for the years between 2005 and 2008 is missing, which possibly could have given a different picture if included. Lundén *et al.* [32] showed that the *T. gondii* prevalence varied between 10% and 45% in the same sheep flock when sampled twice a year for 6 years. This supports the hypothesis that environmental oocyst contamination is fluctuating at least locally, which could be reflected as a varying seroprevalence also in wild boars as in our study.

Due to the rapid expansion of the Swedish wild boar population during the study period and possible time-dependent differences in prevalence, we chose to compare regions only for the years 2010–2011. One region, South Sweden, stood out markedly with a significantly higher seroprevalence (65%, 95% CI 51–77) than all other regions. This difference could not be explained by differences in age distributions since animals from the Småland and the Islands region, located just north of South Sweden, had a similar age distribution but a significantly lower prevalence (45%, 95% CI 38–53, *P* < 0·05). A north–south gradient in *T. gondii* seroprevalence has previously been reported for example in moose and lynx in Sweden [7, 8] and in Finland [33, 34]. These authors suggested that the most likely reason for the low seroprevalence in the far north is a low population density of domestic cats in these sparsely inhabited regions. Although the present study includes only the southern and central parts of Sweden, the recorded differences in seroprevalence might also be due to variations in human and cat populations. Other reasons could be meteorological factors such as precipitation [35] and winter temperatures [36], and geographical conditions such as altitude [37], which have been identified as risk factors for *T. gondii* infections in outdoor cats and wild boars. However, as the present study was based on a convenience sample, far-reaching conclusions concerning the differences found in seroprevalence between regions cannot be made.

A variety of *T. gondii* ELISA systems have been developed and used, sometimes without presenting estimations of diagnostic Se and Sp, making comparisons of test performance difficult. Moreover, commercial serological tests validated for use in defined host species, populations and sample materials are often used to screen entirely different ones, even though test evaluations have indicated that the same test can show different characteristics when applied to other species [16]. Thus, tests should be evaluated before use in new species and populations, and this should be considered when results are interpreted [38]. By estimating test performance for a series of cut-off values we were able to select the most suitable cut-off for our prevalence study. At an OD cut-off of 0·39, the Se and Sp were estimated to 79% and 85%, respectively, and this was regarded as adequate for the purposes of the survey. For diagnostic testing of individual animals, a higher Se and Sp would be preferable.

DAT is considered a reliable serological test for *T. gondii* IgG screening in many animal species [13]. The test is relatively often performed in new species (and animal populations) without a thorough evaluation of test characteristics. In the current study, DAT was evaluated at a single cut-off (1:20), because our primary goal was to retrieve estimates for the ELISA. The test characteristics derived for DAT at this cut-off were close to those obtained in similar studies of domestic pigs [39]. However, which cut-off to use for wild boar samples is a matter of debate. Richomme *et al*. [40] isolated viable parasites in 11/24 wild boars with end-point titres of 1:6 or 1:12, which may indicate recent infections with incipiently increasing antibody titres. This finding was interpreted as 1:6 being a biologically more relevant cut-off than 1:24 (i.e. many false-negative samples). On the other hand, Puvanesuaran *et al*. [41] found a perfect agreement between high serum titres (>1:24) and presence of viable tissue cysts, whereas none of the samples with lower titres (1:6) were positive for tissue cysts. These results indicate that DAT characteristics need to be evaluated (for several cut-offs) when used on novel animal populations such as wild boars.

In the absence of a perfect reference test, the ELISA set up for this study was evaluated using a latent class approach. The Bayesian latent class process for test evaluation has been thoroughly described for chronic *T. gondii* infection in food animals by Gardner *et al*. [39] and has the advantage that estimates of diagnostic Se and Sp could be based on results from naturally infected animals, i.e. the target population of the investigation. In addition, previously collected information on test performances and population prevalences could be included in the model, giving more impartial and precise estimates. The model chosen was based on test results from two potentially dependent tests (ELISA and DAT) and two populations. Because many studies indicate a higher prevalence of *T. gondii* in older animals, subpopulations created here were based on age. However, one assumption in the calculations was that test Se and Sp were equal in both populations, and although age has been suggested as an appropriate factor in this model [39, 42], others have claimed that this is not advisable [43]. One argument is that older individuals may have been exposed to a greater variety of pathogens, which could lead to a difference in immune responses, and consequently, test performance. In our case, the probability of false-positive results may be slightly increased in the adult population (i.e. Sp is lower). If so, the true Se of the test is probably higher than estimated.

This study has demonstrated a high seroprevalence of *T. gondii* in wild boars in Sweden and suggests that wild boar meat is a potential source of *Toxoplasma* infection to a growing group of consumers. Further studies are needed to establish risk factors and efforts must be made to inform the public how to prevent infection.
